# Supplementary material for: Descriptive regression tree analysis of intersecting predictors of adult self-rated health: Does gender matter? A cross-sectional study of Canadian adults
Source: PLoS One. 2023 Nov 14;18(11):e0293976. doi: 10.1371/journal.pone.0293976 (PMC10645330; doi:10.1371/journal.pone.0293976)
Supplement: S1 File — (DOCX) [file pone.0293976.s001.docx]

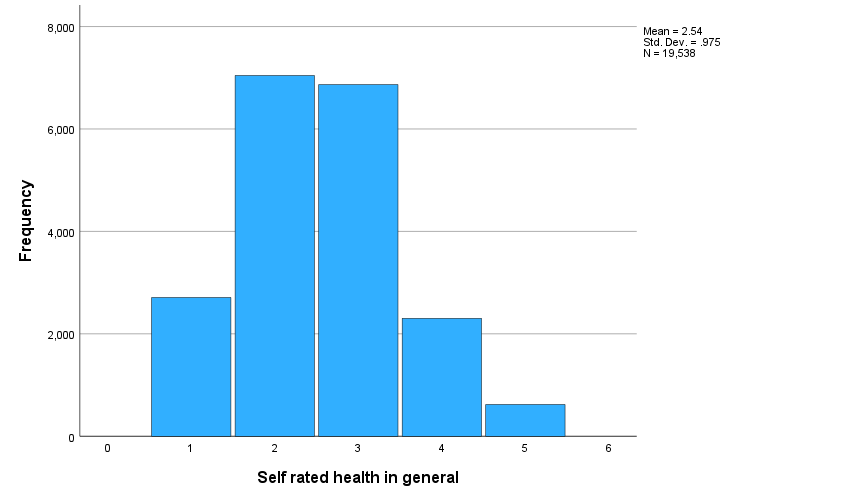


Histogram 1: Frequency distribution of the original scale of self-rated health; the Canadian General Social Survey (GSS) data 2016


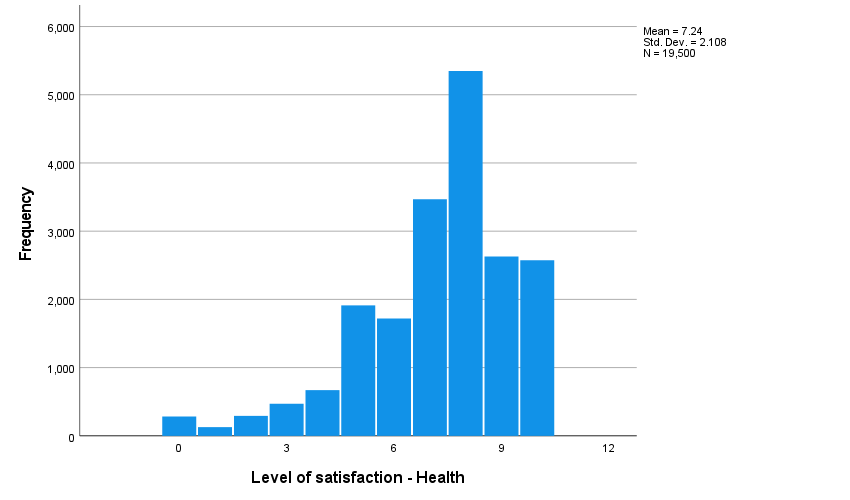


Histogram 2: Frequency distribution the original scale of level of satisfaction with health; the Canadian General Social Survey (GSS) data 2016
