# Supplementary material for: Descriptive regression tree analysis of intersecting predictors of adult self-rated health: Does gender matter? A cross-sectional study of Canadian adults
Source: PLoS One. 2023 Nov 14;18(11):e0293976. doi: 10.1371/journal.pone.0293976 (PMC10645330; doi:10.1371/journal.pone.0293976)
Supplement: Attachment — Submitted filename: Response to Reviewers_SRH_SH_R2.docx [file pone.0293976.s004.docx]

**From:** em.pone.0.860543.6bc3f800@editorialmanager.com <em.pone.0.860543.6bc3f800@editorialmanager.com> **On Behalf Of** PLOS ONE
**Sent:** Wednesday, September 13, 2023 7:41 PM
**To:** Afshin Vafaei <avafaei2@uwo.ca>
**Subject:** PLOS ONE Decision: Revision required [PONE-D-23-10826R1] - [EMID:e4939a41a98b66a8]

PONE-D-23-10826R1

Descriptive regression tree analysis of intersecting predictors of adult self-rated health: does gender matter? A cross-sectional study of Canadian adults

PLOS ONE

Dear Dr. Vafaei,

Thank you for submitting your manuscript to PLOS ONE. After careful consideration, we feel that it has merit but does not fully meet PLOS ONE’s publication criteria as it currently stands. Therefore, we invite you to submit a revised version of the manuscript that addresses the points raised during the review process.

The reviewers have appreciated your efforts to improve your work by implementing their comments. However, before you can recommend publication of your paper, you should take into account the minor comments made by the 3# reviewer in his latest review. Keep in mind that the reviewers have done a really thorough job, and their suggestions have contributed to the improvement of the final paper. 

Please submit your revised manuscript by Oct 28 2023 11:59PM. If you will need more time than this to complete your revisions, please reply to this message or contact the journal office at [plosone@plos.org](mailto:plosone@plos.org). When you're ready to submit your revision, log on to <https://www.editorialmanager.com/pone/> and select the 'Submissions Needing Revision' folder to locate your manuscript file.

Please include the following items when submitting your revised manuscript:

- A rebuttal letter that responds to each point raised by the academic editor and reviewer(s). You should upload this letter as a separate file labeled 'Response to Reviewers'.
- A marked-up copy of your manuscript that highlights changes made to the original version. You should upload this as a separate file labeled 'Revised Manuscript with Track Changes'.
- An unmarked version of your revised paper without tracked changes. You should upload this as a separate file labeled 'Manuscript'.

If you would like to make changes to your financial disclosure, please include your updated statement in your cover letter. Guidelines for resubmitting your figure files are available below the reviewer comments at the end of this letter.

If applicable, we recommend that you deposit your laboratory protocols in protocols.io to enhance the reproducibility of your results. Protocols.io assigns your protocol its own identifier (DOI) so that it can be cited independently in the future. For instructions see: <https://journals.plos.org/plosone/s/submission-guidelines#loc-laboratory-protocols>. Additionally, PLOS ONE offers an option for publishing peer-reviewed Lab Protocol articles, which describe protocols hosted on protocols.io. Read more information on sharing protocols at <https://plos.org/protocols?utm_medium=editorial-email&utm_source=authorletters&utm_campaign=protocols>.

We look forward to receiving your revised manuscript.

Kind regards,

Jordi Gumà, Ph.D.
Academic Editor
PLOS ONE

Journal Requirements:

Please review your reference list to ensure that it is complete and correct. If you have cited papers that have been retracted, please include the rationale for doing so in the manuscript text, or remove these references and replace them with relevant current references. Any changes to the reference list should be mentioned in the rebuttal letter that accompanies your revised manuscript. If you need to cite a retracted article, indicate the article’s retracted status in the References list and also include a citation and full reference for the retraction notice.


[Note: HTML markup is below. Please do not edit.]

Reviewers' comments:

Reviewer's Responses to Questions

**Comments to the Author**

1. If the authors have adequately addressed your comments raised in a previous round of review and you feel that this manuscript is now acceptable for publication, you may indicate that here to bypass the “Comments to the Author” section, enter your conflict of interest statement in the “Confidential to Editor” section, and submit your "Accept" recommendation.

Reviewer #3: (No Response)

2. Is the manuscript technically sound, and do the data support the conclusions?

The manuscript must describe a technically sound piece of scientific research with data that supports the conclusions. Experiments must have been conducted rigorously, with appropriate controls, replication, and sample sizes. The conclusions must be drawn appropriately based on the data presented.

Reviewer #3: Yes

3. Has the statistical analysis been performed appropriately and rigorously?

Reviewer #3: Yes

4. Have the authors made all data underlying the findings in their manuscript fully available?

The [PLOS Data policy](http://www.plosone.org/static/policies.action#sharing) requires authors to make all data underlying the findings described in their manuscript fully available without restriction, with rare exception (please refer to the Data Availability Statement in the manuscript PDF file). The data should be provided as part of the manuscript or its supporting information, or deposited to a public repository. For example, in addition to summary statistics, the data points behind means, medians and variance measures should be available. If there are restrictions on publicly sharing data—e.g. participant privacy or use of data from a third party—those must be specified.

Reviewer #3: Yes

5. Is the manuscript presented in an intelligible fashion and written in standard English?

PLOS ONE does not copyedit accepted manuscripts, so the language in submitted articles must be clear, correct, and unambiguous. Any typographical or grammatical errors should be corrected at revision, so please note any specific errors here.

Reviewer #3: Yes

6. Review Comments to the Author

Please use the space provided to explain your answers to the questions above. You may also include additional comments for the author, including concerns about dual publication, research ethics, or publication ethics. (Please upload your review as an attachment if it exceeds 20,000 characters)

Reviewer #3: Thanks for revising the manuscript and considering and answering my previous comments.

I have four remaining points for a minor revision. After these minor points are addressed I would recommend to accept the paper.

1. Thanks for clarifying within the discussion, that the bivariate analyses of sex and SRH, and sex and SH were performed and both showed significant effects. I feel like these are decisive results in order to draw the conclusion of your paper. I would therefore really appreciate if you could actually report this data in the results section. It should not use up to much space and is important for the overall conclusion of the paper.

Response: We added this sentence to the results section before explaining table 1 which include the composite SRH and SH variable “Before combining SRH and SH variables to generate a composite measure for our outcome, we examined their bivariate relationships with sex. Both showed significant associations according to the results of Chi-square test (p. value for SRH <0.001 and for SH=0.003).”

2. Would it be possible to show the initial distribution (before dichotomizing) of both the SRH and satisfaction with health outcome variables in a supplemental material document. This would surely benefit all readers that are interested in using similar outcome variables and would support your dichotomization decisions.

Response: thank you, a valid point since there is no established cut-off point for dichotomizing (See page 7 of the manuscript). We added these two histograms representing frequency distributions of original scales of SRH and SH as a supplement material to the revised submission.

3. Thanks for clarifying in your response to my question which decision tree algorithm you used. I would recommend to include this one sentence: "we applied the Gini impurity with a minimal improvement set at 0.0001 and equal cost (the full SPSS syntax is available upon request)" into your methods section of the manuscript. It is only one sentence but can really help any readers that would like to use your technique for their analyses. Readers not interested in the techniqual details can always skipp reading the detailed methods section.

Response: Agree, this one sentence contains important information about our analytic method. Added to the ‘statistical analysis’ subsection of *methods* (page 10).

4. I still feel that the sentence "The regression tree correctly classified almost 90% of participants with positive outcomes and resulted in 24 terminal nodes, ten of which were deemed important.”, somehow reports the data one sided, as only the better classification rate (for "positive outcomes") is reported and not the classification rate for participants with poor SRH/SH. You clarified this for me in your response to my question but did not include it in the manuscript.

Response: we reported the 76% overall correct classification in the *methods*. We agree that to avoid a one-sided report of data a more detailed description of misclassification(s) is warranted. We edited the above statement in page 12 to*:* “The regression tree correctly classified 93.3% of participants with positive outcomes and resulted in 24 terminal nodes, ten of which were deemed important. Correct classification for those with negative outcome was 35% yielded a total correct classification of 76%.”

7. PLOS authors have the option to publish the peer review history of their article ([what does this mean?](https://journals.plos.org/plosone/s/editorial-and-peer-review-process#loc-peer-review-history)). If published, this will include your full peer review and any attached files.


If you choose “no”, your identity will remain anonymous but your review may still be made public.


**Do you want your identity to be public for this peer review?** For information about this choice, including consent withdrawal, please see our [Privacy Policy](https://www.plos.org/privacy-policy).

Reviewer #3: **Yes:** Lisa Dandolo

[NOTE: If reviewer comments were submitted as an attachment file, they will be attached to this email and accessible via the submission site. Please log into your account, locate the manuscript record, and check for the action link "View Attachments". If this link does not appear, there are no attachment files.]

While revising your submission, please upload your figure files to the Preflight Analysis and Conversion Engine (PACE) digital diagnostic tool, <https://pacev2.apexcovantage.com/>. PACE helps ensure that figures meet PLOS requirements. To use PACE, you must first register as a user. Registration is free. Then, login and navigate to the UPLOAD tab, where you will find detailed instructions on how to use the tool. If you encounter any issues or have any questions when using PACE, please email PLOS at [figures@plos.org](mailto:figures@plos.org). Please note that Supporting Information files do not need this step.
