# Supplementary material for: Descriptive regression tree analysis of intersecting predictors of adult self-rated health: Does gender matter? A cross-sectional study of Canadian adults
Source: PLoS One. 2023 Nov 14;18(11):e0293976. doi: 10.1371/journal.pone.0293976 (PMC10645330; doi:10.1371/journal.pone.0293976)
Supplement: Attachment — Submitted filename: Reviewer Comments.docx [file pone.0293976.s002.docx]

Dear Authors

I enjoyed reading your manuscript. I think it is a relevant topic for the field. I feel the idea of combining self-rated health with health satisfaction is very interesting and may help overcome the problems of using only self-rated health. I also feel using exploratory classification tree analysis here is a good technique to find subgroups otherwise not identified.

However, I have a few comments and would appreciate if these could be addressed in a revision process.

1. **Major point:** In the **discussion** of the paper the following part is included *“Of note, sex did not emerge as a predictor of the combined outcome of SRH/SH suggesting that adding satisfaction with health corrected for gender differences in interpretation of the meaning of SRH, alone.”* I understand from the background section of the paper, that in previous research sex appeared as a predictor of SRH. However, what I asked myself: is sex a predictor for SRH in this particular population? Did you perform a bivariate analysis for sex and SRH? Was this significant? Did you perform a CRT analysis for only the outcome SRH? Did sex appear as an important variable in this analysis? I feel like adding the results of these analyses to the paper would elevate the manuscript significantly. What if sex was never a predictor of SRH in this population from the beginning. Then the statement in the discussion that *“adding satisfaction with health corrected for gender differences”* cannot be made.
2. **Interchangeable use of the terms “sex” and “gender”.** I noticed that throughout your manuscript, you used the terms “sex” and “gender” interchangeable. However, these two terms do not describe the same concept. It should be clearly defined whether sex or gender is addressed. There is also the possibility of using the term “sex/gender” throughout the manuscript to acknowledge the fact that the two are intertwined as expressed in the embodiment theory. However, if this term is used it has to be explained and described. (See e.g. Hammarström & Annandale 2012 <https://doi.org/10.1371/journal.pone.0034193> and Springer et al. 2012 (<https://doi.org/10.1016/j.socscimed.2011.05.033>)
3. **Dichotomization of outcome variables:** I understand that it makes sense to dichotomize the outcome variables for CRT analysis. However, I would appreciate a better explanation of how it was decided to dichotomize at the chosen cut-off points. E.g. was the cut-off for SRH between good and fair chosen before the distribution of the data was seen, or afterwards based on the distribution. I would actually find it helpful to see the initial distribution too, eg. Did the majority of people categorized as good health actually indicate ‘excellent’, ‘very good’, ‘good’ health. Same for the outcome satisfaction with health, here it is stated that “*This cut-point was selected given the apparent bimodal distribution of responses*.” Is it possible to show this bimodal distribution (maybe in a supplemental material).
4. As sex (or gender) is one of the main focusses of the paper (at least based on the title of the paper) I would expect a clearer explanation of how this variable was measured. It is stated that “*Sex was dichotomous (male/female) and based on participant's self-identification*.” Does this mean that participants could identify as only male or female in the questionnaire (forced choice), or did they have the opportunity to identify as divers, trans, intersex or another category and this was later dichotomized in any way?
5. **CRT analysis:** Why was the maximum tree depth chosen to be 6? Is this based on any prior literature or recommendations? Or were indeed different depths explored and this was the one with the lowest misclassification error? I feel like a depth of 6 is rather high. In other papers performing CRT analysis I have seen trees with less depth in order to retain interpretability.
6. **CRT analysis:** Can some more information be provided on the splitting algorithm used by this particular CRT analysis. There are many different types of classification and regression tree analyses, and I could not clearly understand which algorithm was used (e.g. misclassification error, information gain or Gini impurity).
7. **CRT analysis:** It is stated that “75% correct classifications were sufficient for a reliable and stable tree”. Is there any literature that can suggests that this percentage of correct classifications is acceptable?
8. **Description of results:** I am not sure if I understand the following sentence *“The regression tree correctly classified almost 90% of participants with positive outcomes and resulted in 24 terminal nodes, ten of which were deemed important.”* This means that 90% of participants with good SRH/satisfaction were classified correctly, but from the participants with a bad SRH/satisfaction a lower percentage were classified correctly? Could this percentage also be reported?
9. **Description of results:** Minor point: I had to read the sentence *“…more than 20% different from the whole sample rate that was estimated equal to 68.9%”* a few times till I understood. From the construction of the sentence, I thought there is a sample rate of 68.9%, until I realized that within the whole sample 68.9% rated their SRH/satisfaction as good. Maybe the sentence can be constructed differently.
10. **Description of results:** I was a bit confused by the following two sentences *“Eight nodes enriched for poor SRH/satisfaction with health (between 19.6% and 46.6%) in comparison to the whole survey population. In this context, enriched indicates that the proportion of respondents with positive outcomes (good SRH and high SH) in the specified subpopulation*

*exceeds that among the whole study population (68.9%; node 0, Figure 2)”.* The wording “in this context” indicated for me that in both sentences the same kind of enrichment is described, but in fact in the first sentence the nodes with a lower SRH/satisfaction are described but in the second sentence does nodes with a higher SRH/satisfaction.

11. **Table 1:** Could it be that for the age variable the signs < and > were mixed up? Or where only 28.7 % of the population younger than 65 years?
12. **Table 1:** I was confused by the fact that according to Table 1 amongst people with poor satisfaction or SRH a higher percentage of people (25%) showed a high resilience compared to the 6% high resilience amongst people with a good SRH and good SH. I would have expected the opposite. Could this be discussed?
13. **Table 1:** Similar for alcohol consumption. According to Table 1 people with poor satisfaction or SRH drink less alcohol than people with a good SRH and good SH. I would have expected the opposite. Could this be discussed?
14. **Table 1:** Minor point: Sometimes it is not clear in which line certain numbers belong. One example: in the row “Perceived social class”, do the numbers 224 (2) 399 (7) belong to Lower class or Missing.
15. In the **conclusion** of the paper nothing is mentioned about gender (or sex). I found that to be incoherent with both the title of the paper *“does gender matter?”* and the conclusion part of the abstract “*Combining SRH and SH eliminated sex as a predictor of SRH/SH,…”*
